# Prevalence and clinical characteristics of dry eye disease in community-based type 2 diabetic patients: the Beixinjing eye study

**DOI:** 10.1186/s12886-018-0781-7

**Published:** 2018-05-10

**Authors:** Xinrong Zou, Lina Lu, Yi Xu, Jianfeng Zhu, Jiangnan He, Bo Zhang, Haidong Zou

**Affiliations:** 1 Shanghai General Hospital, Nanjing Medical University, No. 100, Haining Road, Hongkou District, Shanghai, 200080 China; 2 Department of Preventative Ophthalmology, Shanghai Eye Disease Prevention and Treatment Center, No. 380, Kangding Road, Jingan, Shanghai, 200040 China; 3 Department of Ophthalmology, Shanghai General Hospital, Shanghai Jiao Tong University, Shanghai, China; 4 Shanghai Key Laboratory of Fundus Disease, Shanghai, China; 5 Department of Ophthalmology, Fengcheng Hospital, No.9983, Chuannanfeng Road, Fengxian District, Shanghai, 201411 China

**Keywords:** Prevalence, Dry eye disease, Community-based, Type 2 diabetic patients

## Abstract

**Background:**

This study was performed to evaluate the prevalence and clinical characteristics of dry eye disease (DED) in community-based type 2 diabetic patients and to identify the associated factors related with DED.

**Methods:**

A total of 1360 type 2 diabetic patients in the Beixinjing community were randomly selected. All participants were given a questionnaire that assessed basic information and subjective symptoms.DED was diagnosed using the revised Japanese DED diagnostic criteria. All subjects underwent a routine ophthalmic examination, corneal sensitivity test, tear film break-up time(BUT) test, Schirmer I test, fluorescein and lissamine green staining(FL) and fundus photography. Diabetic retinopathy (DR) was graded according to the International severity scale of diabetic retinopathy and diabetic macular edema.

**Results:**

Of the 1360 subjects, 238 (17.5%) were diagnosed with DED. There was a significant association between the presence of DED and higher blood glucose (*P* < 0.001, OR1.240) as well as higher levels of glycosylated hemoglobin HbA1c (*P* < 0.001, OR1.108). Corneal sensitivity was negatively correlated with the prevalence of DED (*P* = 0.02, OR0.973).

**Conclusions:**

The prevalence of DED in this community-based study was 17.5%, which was lower than that observed in hospital-based studies. Diabetic patients with poor metabolic control were more likely to present with DED. A dry eye examination should be added to the routine screening of diabetes.

## Background

Diabetes mellitus is a serious public health problem in many countries. Over the last three decades, the number of diabetic patients worldwide has more than doubled [1]. Diabetes mellitus is a systemic disease that can cause a variety of ocular complications, including diabetic retinopathy, cataracts, glaucoma, keratopathy, and dry eye disease (DED). According to the international dry eye workshop, DED is defined as an abnormality in the quality or quantity of tears or in tear dynamics due to any cause, resulting in ocular discomfort, visual disturbance, decreased tear film stability, and potential damage to the ocular surface [2]. Previous studies have reported an increased prevalence of DED in diabetic patients compared with healthy subjects. Moreover, dry eye symptoms, such as itching, burning, and foreign body sensation, are frequently encountered among diabetic patients [3]. For example, Jin et al. found that type 2 diabetic patients were more likely to suffer from tear film dysfunction [4]. Manaviat et al. reported that the prevalence of DED was 54.3% in type 2 diabetic patients and that the morbidity rate was much higher than that in non-diabetic subjects [5]. They also found that DED was significantly associated with age, sex, duration of diabetes, and diabetic retinopathy. Najafi et al. showed that the prevalence of DED was 27.7% in diabetic patients and that DED showed a significant correlation with glycosylated hemoglobin (HbA1c) [6].

However, all of the published epidemiological studies on DED in type 2 diabetic patients are hospital-based. DED risk factors may differ between hospital-based and community-based populations. Among studies of diabetic patients, hospital-based studies enrolled patients who were in a more serious condition, such as those with a long duration of diabetes and higher level of HbA1C. Until now, it was unknown whether DED was also prevalent in community-based populations. In the present study, we examined the prevalence and clinical characteristics of DED in community-based type 2 diabetic patients in Shanghai, China, and explored the risk factors associated with DED in this group.

## Methods

The study was carried out following the tenets of the Declaration of Helsinki. The Medical Ethics Committee of the Shanghai General Hospital, Shanghai Jiaotong University approved this study protocol, and informed written consent was obtained from all subjects.

### Study design

This community-based cross-sectional study focused on DED among type 2 diabetic patients in the Beixinjing community of Shanghai. The Beixinjing community is in the northwest region of Shanghai and exhibits nationally representative demographic and socioeconomic characteristics. A series of epidemiological investigations have been carried out in this community because of its relatively stable population (43,839 in the 2010 census, of which 11,312 were aged 60 years or older).The social and economic demographics in the Beixinjing community are representative of the middle class in China: the average per capita annual income among urban households is 43,185 yuan(6170 US dollars) [7].

### Diagnostic criteria

Since there is no international diagnostic standard for DED, this study used the revised Japanese DED diagnostic criteria:(1) the presence of dry eye symptoms; (2) presence of either a quantitative or qualitative disturbance of the tear film (BUT ≤5 s or SchirmerItest ≤5 mm); and (3) presence of conjunctivo-corneal epithelial damage (fluorescein staining score ≥ 3 points, Rose Bengal staining score ≥ 3points, or lissamine green staining score ≥ 3 points). A definitive diagnosis of DED was established only if all three criteria were present. Subjects with 0–1 positive criteria were diagnosed as normal [8].

### Diabetic retinopathy (DR)

The DR for each subject was graded based on the worse eye. The grading was performed using the International severity scale of diabetic retinopathy and diabetic macular edema [9].

### Diabetic nephropathy

The urine sample was collected from every subjects to measure urine albumin and creatinine. Microalbuminuria was diagnosed if the albumin/creatinine ratio was greater than 30μg/ mg. And if the ratio was greater than 300 μg/mg, then the subject was identified as macroalbuminuria [6].

### Sampling and exclusion criteria

The reported DED prevalence rate of Chinese adults varied from 7.99 to 21% [10–12]. The formula n = Z^2^(p) (1–p)/B^2^ was adopted to calculate the required sample size via simple random sampling, where Z = 1.96 (with a 95% confidence interval), p is the estimated prevalence, and B is the presupposition error. If it is assumed that *p* = 7.99%, B = 7.99% × 0.25(with a 25% error bound) = 0.019975, and Z = 1.96, then *n* = 708. Using a sample effect coefficient of 1.1 for this study, the estimated response rate was 90%. The calculated number of samples was 865.

From April to September 2016, a community-based study of the prevalence of dry eye in type 2 diabetic patients was performed in the Beixinjing community of Shanghai. Random cluster sampling was used to select the samples used in this study. The clusters were defined geographically based on residential registration data. According to the chronic disease records of the Beixinjing Community Health Service Center, 1503 T2DM residents lived in the 10 randomly selected geographical clusters from total 34 clusters. Type 2 diabetes mellitus was diagnosed according to the criteria of the World Health Organization [13]. The healthy group was aslo selected using random cluster sampling.

Patients with the following ocular disorders that can affect tear production or quality were excluded from the study: (1) eyelid disease: ectropion, entropion, trichiasis, ptosis,eyelid movement disorder caused by facial paralysis; (2) diseases of the conjunctiva: conjunctivochalasis, pterygium; (3)ocular surgeries within 6 months, refractive surgeries within 2 years, history of ocular chemical injury, or use of ocular medications or nutritional tear supplements; and (4) systemic diseases:autoimmune disease:such as Sjogren’s, rheumatoid arthrits,systemic lupus erythematosus. Other systemic diseases: Grave’s disease,Parkinson’s disease.

### Study procedures

At the beginning of the investigation, all participants answered a subjective symptoms questionnaire. The subjective complaints that were assessed included dry eye, itching, burning, foreign body sensation, eye fatigue, lacrimation, and photophobia. If the individual presented with two or more of these symptoms, the subject was classified as having subjective complaints [11, 13]. Next, the participants completed a detailed interview assessing basic information (such as age, gender, nationality, marital status, degree of education, working situation) and past medical histories of systemic diseases (including diabetes, hypertension, rheumatoid arthritis, and cardiocerebrovascular illnesses). The duration of illnesses and medications were noted. The subjects’ height, weight and blood pressure were assessed, then the blood samples and urine samples were collected for the measurement of glucose, glycosylated hemoglobin (HbA1C), serum lipids, and urine albumin and creatinine. The subjects also underwent the following ophthalmological examinations: automatic refractor testing, visual acuity testing(if visual acuity was worse than 0.7, the best-corrected visual acuity was measured), corneal sensitivity testing using a Cochet-Bonnet aesthesiometer (Luneau Ophthalmology, Paris, France), tear-film breakup time (BUT) testing, fluorescein and lissamine greenstaining(FL), SchirmerItest without anesthesia, and slit-lamp examination. In addition, two digital photographs of the fundus were obtained for each eye using a digital 45-μ non-mydriatic retinal camera (non-mydriatic retinal camera; Topcon, Tokyo, Japan). One fundus photograph was centered on the optic nerve head, and the other was centered on the macula.

A Cochet-Bonnet aesthesiometer was used to measure corneal sensitivity The central region of the cornea was tested. An aesthesiometer with a 60 mm long monofilament was placed perpendicularly to the participant’s cornea. The subject was asked whether this touch could be felt when the monofilament was slightly bent. The length of monofilament was recorded as the corneal sensitivity if the subject had felt this touch, and if not, the length of was decreased by 5.0 mm each time until the subject could feel the touch. Three measurements were performed at each monofilament length, and the average length was considered to be the corneal sensitivity at the tested region.

To determine the BUT, a fluorescein strip was placed in the inferior fornix of the subject. Then, the patient was asked to blink several times to distribute the fluorescein across the entire cornea. A cobalt blue filter was used in slit lamp biomicroscopy to record the interval between the last blink and first appearance of a dry spot on the cornea. This measurement was repeated three times, and the average value was calculated. Values shorter than 5 s are diagnostic for DED.

For the Schirmer I test, a Schirmer strip was inserted into the inferior fornix beneath the temporal lid margin without anesthesia. After 5 min, the Schirmer strip was removed and the scale of wetness was measured. Patients were informed not to rotate their eyes. Values lower than 5 mm imply a diagnosis of DED.

To evaluate the FL of the ocular surface, the eye was divided into three equal compartments: the cornea, the nasal conjunctiva and the temporal conjunctiva. The maximum staining score for each area was 3points, and the maximum overall staining score was 9 points [8].

### Statistical methods

IBM-SPSS version 23 was used for statistical analysis. The chi-square test, independent paired t test and Pearson correlation analyses were performed. A *P* value < 0.05 was accepted as statistically significant.

A binary logistic regression analysis was performed using the presence of DED as the dependent parameter. The independent variables included those parameters that were significantly associated with DED in univariate analysis.

## Results

The Diabetes Group was well matched with the Healthy Group in terms of age and sex. The subjects in the Diabetes Group ranged in age from 31 to 95 years, while those in the Healthy Group were 47 to 94 years. (Table 1).

**Table 1:** Demographic characteristics of the diabetes group and healthy group

Characteristic	Sub-group	Diabetes Group	Healthy Group	Statistic Value	*P* Value
Age(years)		68.91 ± 8.86	68.31 ± 8.09	3.36	0.09
Gender	Female	853	690	2.92	0.09
	Male	507	354		

The Diabetes Group had a significantly higher BMI, blood glucose, triglyceride and total cholesterol than the Healthy Group.However,there was no significant difference in systolic blood pressure and diastolic blood pressure between the two groups. The tear film function and corneal sensitivity parameters were significantly different between the diabetic and healthy subjects. The corneal sensitivity in the Diabetes Group was much lower than that in the Healthy Group. Both the BUT and SchirmerItest scores were significantly lower in the Diabetes group. The diabetic patients had much more unstable tear film than the healthy subjects (Table 2).

**Table 2:** Clinical characteristics of the diabetes group and healthy group

Factor	Diabetes Group	Healthy Group	Statistic Value	*P* Value
Corneal sensitivity (mm)	54.27 ± 10.64	58.11 ± 4.57	−10.91	0.00
BUT(s)	3.87 ± 2.52	5.65 ± 2.64	−16.81	0.00
Schirmer I test (mm)	8.95 ± 6.24	9.49 ± 6.62	−2.05	0.00
FL	0.60 ± 1.04	0.30 ± 0.97	8.91	0.00
BMI (kg/m^2^)	25.53 ± 3.89	24.16 ± 3.71	2.73	0.00
Glucose (mmol/L)	7.92 ± 2.51	6.66 ± 2.74	11.72	0.00
Triglyceride (mmol/L)	1.81 ± 1.32	1.32 ± 1.86	7.55	0.00
Total cholesterol (mmol/L)	5.10 ± 1.17	4.84 ± 0.84	6.08	0.00
SBP (mmHg)	138.27 ± 21.88	134.83 ± 24.48	1.52	0.13
DBP (mmHg)	75.44 ± 18.15	74.19 ± 14.94	1.80	0.07

*BUT* tear-film break up time, *FL* fluorescein and lissamine green staining, *BMI* Body Mass Index, *SBP* systolic blood *pressure, DBP* diastolic blood *pressure*

A total of 238(17.5%) out of 1360 type 2 diabetic subjects were definitively diagnosed with DED. In the Healthy group, DED was detected in 62 participants (5.9%). There was a significant difference in the prevalence of DED between the two groups (χ^2^ = 72.25,*p* < 0.001). A total of 551 subjects were not in conformity with the DED or non-DED diagnostic criteria, not included in the statistical analysis.

In the Pearson Correlation analysis, age was negatively correlated with BUT and corneal sensitivity (*P* < 0.05) and positively correlated with FL (*P* < 0.05).The diabetic duration was not correlated with any of the tear film parameters. The HbA1C was negatively correlated with BUT (*P* < 0.05) and positively correlated with FL (*P* < 0.05). The female subjects had shorter BUT,lower SchirmerItest scores and better corneal sensitivity than the males (Tables 3 and 4).

**Table 3:** Pearson correlation analysis of tear film function and contributing factors in the Diabetic Group

Factor		BUT	SchirmerItest	FL	Corneal Sensitivity
Age	Pearson Correlation	−.104*	.080	.157**	−.246**
	Sig. (2-tailed)	.015	.062	.000	.000
Diabetic duration	Pearson Correlation	.004	−.003	−.072	−.004
	Sig. (2-tailed)	.935	.955	.111	.933
HbA1C	Pearson Correlation	−.111**	.006	.155**	−.051
	Sig. (2-tailed)	.009	.880	.000	.231

*BUT* tear-film break up time, *FL* fluorescein and lissamine green staining, *HbA1C* glycosylated hemoglobin, * *P*<0.05, ** *P*<0.01

**Table 4:** The tear film function of different gender in the Diabetic Group

Factor	Sub-groups	*n*	BUT(s)	Schirmer I test	FL	Corneal Sensitivity
Gender	Female	896	3.63 ± 2.10	8.67 ± 5.89	0.59 ± 0.98	55.51 ± 10.21
	Male	464	4.33 ± 2.70	9.59 ± 6.42	0.62 ± 1.16	53.64 ± 10.80
Statistic Value			−5.27	−2.67	− 0.49	3.14
*P* value			0.00	0.01	0.62	0.00

*BUT* tear-film break up time, *FL* fluorescein and lissamine green staining

The single factor analysis in diabetic DED patients detected significant variables, including age, gender, corneal sensitivity, blood glucose, HbA1C, diabetes duration, and total cholesterol, as shown in Table 5. No correlation was found between diabetic retinopathy or diabetic nephropathy classification and the prevalence of DED. A dry eye diagnosis was considered to be the dependent variable. The independent variables included age, gender, corneal sensitivity, blood glucose, HbA1C, diabetes duration, and total cholesterol. In the binary logistic regression analysis, the prevalence of DED was significantly associated with a higher glucose (*P* < 0.001, OR1.240) and higher HbA1C (*P* < 0.001, OR1.108). The corneal sensitivity was inversely correlated with the prevalence of DED (*P* = 0.02, OR0.973), indicating that the higher the corneal sensitivity, the lower the prevalence of DED.

**Table 5:** Demographic and clinical characteristics of DED and non-DED subjects among the 1360 community diabetic subjects

Factors	Groups	DED	Non-DED	Statistic Value	*P* Value
Total Number		238	571		
Age(years)		68.48 ± 8.55	71.30 ± 8.45	4.31	0.00
Gender	Female	157	417		
	Male	81	154	4.07	0.04
Education	Illiterate	18	56		
Level	Primary school	29	87		
	Junior high school	66	142		
	Senior high school	68	189		
	Junior College	32	63		
	Bachelor’s degree or higher	25	34	9.24	0.10
Duration of diabetes(years)		9.45 ± 6.24	8.01 ± 6.69	−2.84	0.00
Corneal sensitivity(mm)		52.05 ± 12.52	54.76 ± 10.11	3.23	0.00
BUT(s)		2.70 ± 0.88	5.12 ± 2.99	12.26	0.00
Schirmer I test (mm)		8.62 ± 6.07	9.61 ± 6.28	2.13	0.03
FL		3.74 ± 1.24	0.03 ± 0.20	−69.42	0.00
BMI (kg/m^2^)		25.50 ± 4.45	25.47 ± 3.97	−0.09	0.92
Glucose (mmol/L)		8.58 ± 2.55	7.33 ± 2.20	−6.61	0.00
HbA1C (%)		7.64 ± 1.55	7.05 ± 1.37	−5.10	0.00
Triglyceride (mmol/L)		1.65 ± 1.22	1.81 ± 1.98	1.16	0.25
Total cholesterol (mmol/L)		5.02 ± 1.05	5.14 ± 1.20	1.34	0.02
DR	Normal	245	407		
	Mild NPDR	5	43		
	Moderate NPDR	19	101		
	Severe NPDR	3	15		
	PDR	3	5	35.67	0.00
Diabetic nephropathy	Normal	391	168		
	microalbuminuria	138	47		
	macroalbuminuria	42	23	2.66	0.26

*BUT* tear-film break up time, *FL* fluorescein and lissamine green staining, *BMI* Body Mass Index, *HbA1C* glycosylated hemoglobin, DR:diabetic retinopathy, *NPDR* nonproliferative diabetic retinopathy, *PDR* proliferative diabetic retinopathy

## Discussion

To the best of our knowledge, this is the first community-based study of DED in type 2 diabetics. In this study, the prevalence of definitive DED in type2 diabetics was 17.5%, which is much greater than that in the healthy group, but lower than in previously published hospital-based epidemiology studies. First, different diagnostic criteria might affect the prevalence of DED reported in the present and previous studies. For example, Manaviat et al. performed Schirmer and BUT tests and utilized the criterion of one positive test to establish the diagnosis in type 2 hospital-based diabetic patients [5]. Najafi et al. used tear osmolarity values (with a cutoff value of 308 mOsm/L) to diagnose DED in their study [6]. Fuerst et al.showed that 52% of their hospital-based diabetic patients had at least mild DED based on OSDI scores(ranging from 13 to 22) [14]. Second, but more importantly, the poor metabolic status of the hospital-based diabetic patients might also contribute to this difference. For example, HbA1C(%) in one hospital-based study was 8.2 ± 2.2, which was higher than that in the present study [14]. Another significant difference between a hospital-based study and community-based study is the duration of diabetes, which is a well-known correlate of DED. In the study by Manaviat et al., the mean duration of diabetes was 11.48 ± 7.4 years in 108 patients with DED and 9 ± 6.5 years in subjects without DED [5]. However, in our community-based study, the diabetes duration was 9.45 ± 6.24 years in DED patients and 8.01 ± 6.69 years in non-DED patients. Both were much shorter than that in the hospital-based study. Therefore, the results of this study provided novel information.

In comparison to the Healthy Group, the Diabetic Group had decreased BUT, Schirmer’s test values, and corneal sensitivity and increased FL, in accordance with previous studies [14–17]. In diabetes, damage to the microvasculature feeding the lacrimal gland together with autonomic neuropathy of the lacrimal gland, both of which occur early in the course of diabetes, may contribute to impaired function of the gland [3, 18]. Additionally, sorbitol accumulation within cells can lead to cellular edema and dysfunction, which causes lacrimal gland damage and dysfunction and decreased tear secretion [19]. Decreased corneal sensitivity is a clinical manifestation of diabetic keratoplasty. A possible mechanism might be that diabetic keratoplasty causes lesions in the corneal epithelial membrane and alters tear protein expression, then tear film function changed and that can lead to DED [20]. Furthermore, reduced corneal sensation can also lead to a reduced blink rate and increased tear evaporation [21]. These potential mechanisms induced DED in the diabetic patients.

The BUT value was found to decrease gradually with increasing age, and the BUT of women was shorter than that of men in the Diabetic Group. However, in contrast to previous studies, age and gender were not found to be relative risk factors for DED in the diabetic group. Possible explanations are that the subjects enrolled in this study were too old (68.91 ± 8.86 years) and that the proportion of male participants in this study was too low. Similarly, Li et al. reported no difference in prevalence of DED between men and women over the age of 60 [10]. The probable cause is reduced tear secretion with aging, or age-related DED, which can affect all exocrine glands in the elderly.

HbA1C is a marker of the average blood glucose level over the 2–3 months prior to measurement. In this study, blood glucose and HbA1C were positively related to the DED prevalence. This result was in accordance with other reports [5, 6, 22]. There are several potential mechanisms for this correlation. Firstly, diabetic patients have an increased level of glucose in their tears, which can increase the expression of advanced glycation end-product(AGE)-modified proteins [23]. The combination of AGEs and receptor for advanced glycation end(RAGE)-products induces a large number of reactive oxygen species (ROS) in corneal epithelial cells, activating downstream signal transduction pathways. The oxidative damage of corneal epithelial cells may result in decreased corneal sensitivity and a delay in wound healing [24]. Secondly, hyperglycemia leads to hyperosmolarity of the tear film and the ocular surface epithelial cells, activating a series of inflammatory responses. Inflammatory cytokines, such as tumor necrosis factor-alpha (TNF-alpha) and matrix metalloproteinase-9 (MMP-9) have been demonstrated to be involved in the pathogenesis of DED [25].Thirdly, hyperglycemia has also been confirmed to lead to histological alterations in the lacrimal gland. Therefore, diabetes-induced oxidative stress plays an important role in DED [26].

Previous studies have examined the relationship between DED and DR [6, 13]. For example, in a study by Ozdemir et al., the BUT score was lower in participants who had retinal laser treatment than in those who did not have this treatment. This study also showed that subjects with PDR had a lower BUT score compared to subjects with NPDR [13]. However, in our study there was no relationship between the DED prevalence and the DR classification after binary logistic regression analysis. In accordance with Najafi et al. ‘s study, there was no relationship between the diabetic nephropathy stratification and the prevalence of DED [6]. There may be an indirect correlation between DED and DR, and also between DED and diabietic nephropathy, because all those conditions were more likely to occur in diabetic patients with poor metabolic control.

Our study has some limitations. Firstly, tear osmolarity measurement was not included in dry eye diagnosis. It is important for the diagnosis of Dry Eye, however, it is not widely used in China. Further more, it’s difficult to carry out tear osmoloarity measurement which takes a long time and costs high in such a community-based epidemiological investigation. Secondly, the subjects in this study were of old age. The aging of the population is a growing and inevitable social problem. According to census data, 30.2% of the Shanghai population was over the age of 60 at the end of 2015 (the city population was 14.4297 million people). Women were more likely to participate in our study than men, and the gender ratio was suboptimal. Thirdly, meibomian gland function was not included in our study. Since dry eye is associated with meibomian gland dysfunction, future studies should take this aspect into consideration.

## Conclusions

In conclusion, DED prevalence was significantly higher in diabetic than healthy subjects, and the overall prevalence of DED was lower in our community-based study than in previous hospital-based studies. Moreover, diabetic patients with poor blood glucose control were more likely to suffer from DED. Therefore, DED testing should be added to the routine screening of diabetes.

## Abbreviations

AGE: Advanced glycation end-product
BMI: Body Mass Index
BUT: Tear-film break up time
DBP: Diastolicblood pressure
DED: Dry eye disease
DR: Diabetic retinopathy
FL: Fluorescein and lissamine green stain
HbA1C: Glycosylated hemoglobin.
NPDR: Nonproliferative diabetic retinopathy
PDR: Proliferative diabetic retinopathy
RAGE: Receptor for advanced glycation end-products
ROS: Reactive oxygen species
SBP: Systolic blood pressure
